# Synthesis and Biological Evaluation of Delavayin-C

**DOI:** 10.4103/0250-474X.49137

**Published:** 2008

**Authors:** Nirmala V. Shinde, M. Himaja, S. K. Bhosale, M. V. Ramana, D. M. Sakarkar

**Affiliations:** Department of Pharmaceutical Chemistry, N. G. S. M. Institute of Pharmaceutical Sciences, Nanthoor, Mangalore-575 005, India

**Keywords:** Cyclic peptide, delavayin-C, antibacterial, antifungal, p-nitrophenylester method

## Abstract

The synthesis of a cyclic heptapeptide, delavayin-C, cyclo(gly-tyr-tyr-tyr-pro-val-pro) is described. The structure of this compound was established on the basis of analytical IR, ^1^H NMR and FAB mass spectral data. The antibacterial and antifungal activities of this peptide are also described.

Cyclic peptides were found to exhibit various biological activities like antibacterial, antifungal, anthelmintic, insecticidal, antineoplastic, antitumor, antiinflammatory activities^1^–^6^. Keeping in view of the significant biological activities exhibited by various cyclic peptides, as a part of ongoing study, an attempt was made towards the synthesis of a cyclic heptapeptide, delavayin-C, cyclo(gly-tyr-tyr-tyr-pro-val-pro), which was isolated from the roots of *Stellaria delavayi* and belongs the family *Cariophyllaceae*^7^. The synthesized compound was further subjected to antibacterial activity against *Bacillus subtilis, Staphylococcus aureus, Escherichia coli* and *Psuedomonas aeruginosa* and antifungal activities against *Candida albicans*.

The synthesized compound has shown moderate antibacterial and antifungal activity comparable with the standard drug benzyl penicillin and standard antifungal agent fluconazole, respectively. Spectral interpretation and elemental analysis was done for the synthesized compound for structural elucidation.

In order to carry out the total synthesis of cyclic peptide, cyclo(gly-tyr-tyr-tyr-pro-val-pro), it was disconnected into three dipeptide units, Boc-gly-tyr-OMe 1, Boc-tyr-tyr-OMe 2, Boc-pro-val-OMe 3 and a single amino acid methyl ester hydrochloride unit, pro-OMe-HCl 4. The required dipeptides were prepared by coupling Boc amino acids with the respective amino acid ester hydrochlorides using DIPC, CHCl_3_ and N-methyl morpholine according to Bondanszky^8^ procedure with suitable modifications. The Boc-group of the dipeptide 2 was removed by using trifluoroacetic acid and the ester group of dipeptide 1 was removed by using LiOH. The deprotected units were then coupled to get a tetrapeptide Boc-gly-tyr-tyr-tyr-OMe 5. Similarly, the dipeptide 3 was coupled with single amino acid methyl ester hydrochloride unit, pro-OMe HCl 4 after appropriate deprotection to get a tripeptide Boc-pro-val-pro-OMe 6. The resulting tetrapeptide and tripeptide was then coupled together by using DIPC, NMM and CHCl_3_ to get a linear heptapeptide Boc-gly-tyr-tyr-tyr-pro-val-pro-OMe 7. Finally cyclisation of this linear heptapeptide was carried out by p-nitrophenyl ester method. The intermediates and the final product were purified by recrystallisation from CHCl_3_. The retrosynthetic analysis of peptide is shown in the Scheme 1

**Scheme 1:**
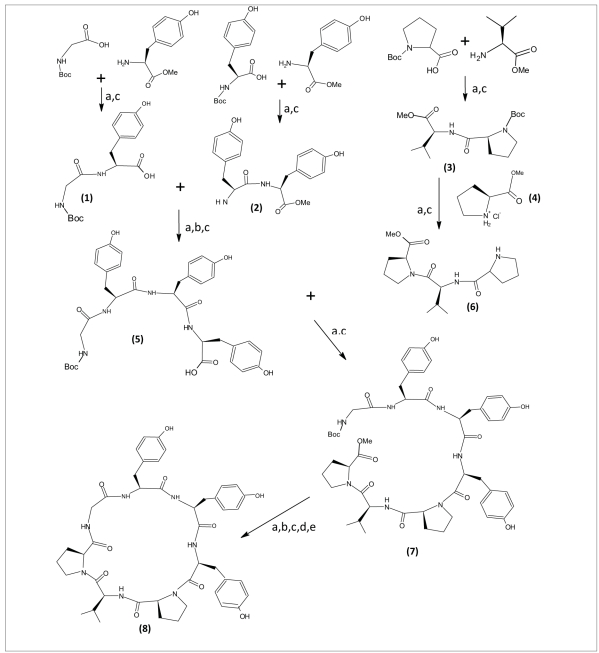
Synthetic route for the synthesis of delavayin-C. a= DIPC, NMM, CHCl_3_, RT, 24 h, b= TFA, NMM, RT, 1 h, c= LiOH, THF:H_2_O (1:1), reflux, 15 mins, d= pnp-, CHCl_3_, RT, 12 h, e= NMM, CHCl_3_, 0°C, 7 d.

The newly synthesized compound was analyzed for C, H, N, and O by elemental analysis and structure was confirmed by IR, ^1^H NMR and FAB mass spectral analysis. The characteristic IR absorption bands of -CO-NH- moiety was present in the cyclised product. The NMR spectrum of cyclised product clearly indicates the presence of all respective amino acid moieties. Furthermore, the mass spectrum of this cyclic heptapeptide showed a molecular ion peak at m/z 840, which corresponds to molecular formula C_44_ H_53_ O_10_ N_7_.

The synthesized cyclic heptapeptide was screened *in vitro* for its antibacterial and antifungal activity by using disc diffusion method and tube dilution technique. The antibacterial activity was determined against four bacterial species (*B. subtilis, S. aureus, E. coli* and *P. aeruginosa*) and antifungal activity against *Candida albican*. In the disc diffusion method, the activity studies were carried out according to modified Kirby-Bauer method^9^. Benzyl penicillin and fluconazole were used as standards against bacterial and fungal strains, respectively at a concentration of 50 μg/ml. Nutrient broth and Sabourds agar were used as a medium and dimethylformamide (DMF) was used as a solvent control for carrying out the activity. After preparation of the disc, allowed to stand for 24 h at 37°. The zone of inhibition, observed around the disks after incubation, was measured. The synthetic peptide has shown moderate activity against *B. subtilis* and *S. aureus* (gram positive bacteria) and less activity against *E. coli* and *P. aeruginosa* (gram negative bacteria) when compared with standard drug benzyl penicillin. The compound has also shown moderate inhibition of growth against *Candida albicans*.

Compound inhibiting growth of microorganisms was further tested for minimum inhibitory concentration (MIC). A solution of the compound was prepared in DMF and a series of doubling dilutions prepared with sterile pipettes. To each of a series of sterile stoppered test tubes, a standard volume of nutrient broth medium was added. A control tube containing no antimicrobial agent was included. The inoculum consisting of an overnight broth culture of microorganisms was added to separate tubes. The tubes were incubated at 37° for 24 h and examined for turbidity. The tube with highest dilution showing no turbidity was the one containing compound with MIC. Screening data of antibacterial and antifungal activity revealed that the synthetic peptide is found to be active. The results are shown in Tables 1 and 2.

**TABLE 1:** ANTIMICROBIAL ACTIVITY BY USING DISC DIFFUSION METHOD

Name of the compound	Diameter of zone of inhibition (mm)				
	
	*S. auereus*	*B. Subtilis*	*P. aeruginosa*	*E. coli*	*C. albicans*
Compound	21	15	11	10	18
Benzyl Penicillin	25	15	17	16	-
Fluconazole	-	-	-	-	20
DMF	-	-	-	-	-

− indicates no activity. Both test compounds and standard were tested at 50 μg/ml.

**TABLE 2:** MINIMUM INHIBITORY CONCENTRATION FOR ANTIMICROBIAL ACTIVITY

Organism used↓	Presence or absence of growth						
	
	concentration of the compound (μg/ml) ↓						
	
	100	50	25	12.5	6.25	3.13	1.56
*S. auereus*	-	+	+	+	+	+	+
*B. subtilis*	–	–	+	+	+	+	+
*P. aeruginosa*	–	+	+	+	+	+	+
*E. coli*	–	+	+	+	+	+	+
*C. albicans*	–	–	+	+	+	+	+

(+) indicates presence of growth (no activity)

Melting points were taken in open capillary tubes and are found to be uncorrected. IR spectra was recorded on Jasco FTIR 5300 IR spectrometer (in CHCl_3_) and the chemical shift values are reported as values as Vmax (cm^−1^). ^1^ H NMR spectra was recorded on Brucker AC NMR spectrometer (300 MH_Z_ in CDCl_3_) and the chemical shift values are reported as values in ppm relative to TMS (δ=0) as a internal standard. FAB mass spectra were recorded on a Joel SX 102/DA-6000 Mass Spectrometer using xenon as a carrier gas. TLC was done to check the progress of reaction by using silica gel-G plates. All the compounds gave satisfactory elemental analysis for C, H, N and O.

The dipeptides 1 and 2 were used for the preparation of a tetrapeptide Boc-gly-tyr-tyr-tyr-OMe (5). The tripeptide Boc-pro-val-pro-OMe (6) was prepared by coupling a dipeptide Boc-pro-val-OMe (3) with pro-OMe HCl (4) unit. The resulting tetrapeptide and tripeptide were coupled by using DIPC and N-methyl morpholine (NMM) to obtain a linear heptapeptide Boc-gly-tyr-tyr-tyr-pro-val-pro-OMe (7). Cyclisation of this linear heptapeptide was carried out by using p-nitrophenyl ester method^10^. The ester group of the linear segment was removed with LiOH and the p**-**nitrophenyl ester group was introduced using the following procedure, The Boc***-***peptide carboxylic acid (1.5 mmol) was dissolved in CHCl_3_ (15 ml) at 0°. Then p***-***nitrophenol was added (0. 27 g, 2 mmol), and stirred for 12 h at room temperature. The reaction mixture was filtered and the filtrate was washed with NaHCO_3_ solution (10%) until excess of p***-***nitrophenol was removed and finally washed with 5% HCl (5 ml) to get Boc***-***peptide***-***pnp***-***ester.

To the above Boc***-***peptide***-***pnp***-***ester (1.2 mmol) in CHCl_3_ (15 ml), CF_3_COOH (0. 274 g, 2. 4 mmol) was added, stirred for 1 h at room temperature and washed with 10% NaHCO_3_ solution. The organic layer was dried over anhydrous Na_2_SO_4_. To the Boc***-***deprotected peptide***-***pnp***-***ester in CHCl_3_ (15 ml), N-methylmorpholine (1.4 ml, 2 mmol) was added and kept at 0° for 7 d. The reaction mixture was washed with 10% NaHCO_3_ until the byproduct p***-***nitrophenol was removed completely and finally washed with 5% HCl (5 ml). The organic layer was dried over anhydrous Na_2_ SO_4_. Chloroform and pyridine were distilled off to get the crude product of cyclized compound, which was then recrystallized from CHCl_3_/n***-***hexane.

Physical state was found to be semisolid mass, molecular formula is C_44_H_53_O_10_N_7_ with a molecular weight of 839. R_f_ value was found to be 0.60 in the solvent system, chloroform:methanol:water (5:3:2). IR data is 3676.4 (OH stretch), 3293.1 (NH stretch), 3017.8 (Arom-CH stretch), 2935.2 (aliph-CH stretch), 2857.8 (aliph-CH stretch), 1658.7 (C=O stretch of amide), 1530.4 (OH-bend) 1451.5 (NH bend) cm^−1^. ^1^H NMR data was δ 10.3 (3H, d, NH), 8.1 (2H, d, NH), 7.65-6.7 (12H, m, Arom-H), 4.9 (1H, d, α-H), 4.7 (1H, d, α-H), 4.55 (2H, m, α-H), 4.4 (1H, m, α-H), 4.2 (1H, m, α-H), 4.0 (1H, m, α-H), 3.9 (1H, m, α-H), 3.7 (4H, m, NCH_2_ of Pro), 3.5 (10H, m, β-CH_2_ of tyr and β-CH_2_ of pro), 2.3 (1H, m, β-H of val), 0.95 (6H, d, (CH_3_)_2_ of val. Molecular ion peak observed at m/z 840 corresponds to the molecular formula C_44_H_53_O_10_N_7_. C: 63.1 (62.92)%, N: 12.16 (11.67)%.

Results of biological activity were shown in Tables 1 and 2. The newly synthesized compounds showed significant antibacterial activity against gram positive bacteria in comparison to the standard drug benzyl penicillin. It has also shown moderate antifungal activity in comparison with the standard drug fluconazole.
